# Navigating microbiome variability: implications for research, diagnostics, and direct-to-consumer testing

**DOI:** 10.3389/fmicb.2025.1580531

**Published:** 2025-04-10

**Authors:** Lorenzo Drago

**Affiliations:** ^1^UOC Laboratory of Clinical Medicine With Specialized Areas, MultiMedica Scientific and Technological Pole, MultiMedica (IRCCS), Milan, Italy; ^2^Clinical Microbiology and Microbiome Laboratory, Department of Biomedical Sciences for Health, University of Milan, Milan, Italy

**Keywords:** microbiome variability, diagnostics, direct-to-consumer testing, standardization, *in vitro* diagnostics (IVD), *Firmicutes*-to-*Bacteroidetes* ratio

## Background

The article by Porcari et al. (2025) addresses an area of growing interest: the potential of microbiome testing in medicine. As microbiome research has evolved, its application in diagnostics has attracted increasing attention. This topic is timely and highly relevant in the context of modern healthcare.

The article provides an important discussion on the current state of microbiome testing, highlighting the need for standardization and regulation. It highlights the gap between the growing interest in microbiome diagnostics and the lack of robust clinical evidence to support its use. This assessment identifies areas that need further research and regulation, but reaching definitive conclusions in my opinion remains a challenge given this complexity.

What is particularly appreciated is the Multidisciplinary Expert Panel suggesting a comprehensive approach to understand the clinical implications of microbiome testing. This ensures that recommendations are based on expertise in microbiology, healthcare, and regulatory bodies.

However, the article should explore some key aspects in more depth. First, the evidence supporting the clinical utility of microbiome diagnostics is scant, and this could limit the applicability of any recommendations. The emphasis on regulation, or striving for claims at all costs, could overshadow the need for robust clinical data.

Regulators, often slow to adapt to new discoveries, could further delay innovation. Microbiome composition is influenced by a multitude of factors, including diet, medication, time of day, and environmental exposures. These dynamics not only vary between individuals but also fluctuate within the same individual under different conditions, making it difficult to draw consistent conclusions.

In this correspondence, I'd like to explore the implications of microbiome variability for research and diagnostics, with a particular focus on standardization, regulatory frameworks, and the risks of direct-to-consumer (DTC) microbiome testing.

## The challenge of microbiome variability

Standardizing practices for microbiome testing is a noble goal, but the implementation could face significant barriers, such as variations in healthcare systems, costs, and the complexity of microbiome science itself. Translating a theoretical framework into practical is a very challenging goal. Variability is an intrinsic feature of the microbiome. Factors such as dietary habits, medication use, and circadian rhythms can significantly alter microbial composition within an individual (Britton et al., 2024). These fluctuations complicate efforts to develop diagnostic tools based on microbiome profiles and highlight the need for a more reproducibility and consistency in research methods.

## Risks of direct-to-consumer testing

The article mentions at the increasing number of commercial providers offering direct-to-consumer microbiome tests, which may lead to potential misuse or misinterpretation of results. However, the article it would be necessary to provide in-depth analysis or solutions to the complex issue of consumer-level testing. It should be important to address the ethical implications of such tests and the risk of patients misusing or misunderstanding the results. DTC microbiome tests, while marketed as tools for personalized health insights, often fail to account for the microbiome's variability (Hoffmann et al., 2024). These tests lack standardization and are prone to misinterpretation by consumers. This can lead to inappropriate health decisions or unwarranted anxiety. Clear guidelines and robust public education are critical to mitigating these risks.

## The need for standardization in diagnostics (sample collection and IVD test)

Establishing Best Practices should be a very promising and challenging goal. This is crucial for ensuring the reliability, validity, and utility of microbiome diagnostics in clinical settings. Standardization could also help minimize errors, increase reproducibility, and build trust in these tests. This includes two relevant steps often neglected: harmonizing protocols for sample collection, processing, and analysis and the use of *In Vitro* Diagnostic (IVD)-certified tests, which follow strict quality control measures, represent an important step toward improving reproducibility and trust in microbiome-based diagnostics (Vandeputte et al., 2017). Requiring IVD tests may indeed ensure that microbiome testing meets high standards of performance, reducing the risk of false positives, false negatives, and errors.

In addition, reliable microbiome testing depends on standardized sample collection methods. Variables such as timing, storage conditions, and the number of samples collected significantly affect test outcomes (Vogtmann et al., 2017). For instance, collecting multiple samples over time or from different body sites may be necessary to capture a representative microbiome profile.

We should then respond to these questions: when and how to collect biological samples, but above all how many samples. Considerations like sterile collection tools, timing of sample collection in relation to food intake or medication, proper storage (e.g., freezing, refrigeration) to preserve microbial DNA or RNA integrity, collection of baseline samples vs. samples taken during or after treatment, should be further discussed in order to standardize this preclinical phase.

## Dedicated laboratories to perform the test

Performing a microbiome test in a hospital or well-organized laboratory is essential because these settings provide specific space and dedicated facilities tailored for such testing. Hospitals and accredited labs are designed with specialized equipment and environments to prevent contamination, ensuring the integrity of the sample, the accuracy and traceability of the results. They also offer controlled environments, such as sterilized rooms and temperature-regulated storage, which are crucial for handling sensitive microbiome samples. Additionally, the presence of trained personnel and advanced diagnostic tools in these facilities ensures reliable analysis and interpretation, which might not be available in less specialized locations. Specifying and better outlining which laboratories can be dedicated to this and what requirements they must have is a fundamental position to define. The final methodology to be used is highly demanding: short amplicon 16S rRNA gene sequencing is currently the method of choice for microbiome test, although in this method certain bacterial genera were found to be underrepresented or even missing taxonomically (Abellan-Schneyder et al., 2021). However, comparative studies on differences in procedures are scarce and a definitive statement should be further discussed.

## The misuse of simplistic metrics

The *Firmicutes*-to-*Bacteroidetes* ratio is a commonly used metric in microbiome research (paramount of references have used per years this parameter in their studies and evaluations), but its oversimplification risks overlooking the complexity of microbial ecosystems. While useful in certain contexts, relying solely on broad metrics can lead to misleading interpretations (Mirzayi and Renson, 2021). A multidimensional approach is necessary to accurately characterize gut microbiota dynamics and should be further clarified and discussed.

## Conclusions

The variability and complexity of the microbiome present significant challenges for its integration into clinical diagnostics. By prioritizing standardization, regulatory frameworks, and consumer education, we can ensure that microbiome-based diagnostics are both reliable and clinically meaningful. Addressing these gaps will pave the way for a more accurate and effective application of microbiome science in healthcare.
